# Hijacking of the Ubiquitin/Proteasome Pathway by the HIV Auxiliary Proteins

**DOI:** 10.3390/v9110322

**Published:** 2017-10-31

**Authors:** Tanja Seissler, Roland Marquet, Jean-Christophe Paillart

**Affiliations:** Université de Strasbourg, CNRS, Architecture et Réactivité de l’ARN, UPR 9002, IBMC-15 rue René Descartes, F-67000 Strasbourg, France; t.seissler@ibmc-cnrs.unistra.fr (T.S.); r.marquet@ibmc-cnrs.unistra.fr (R.M.)

**Keywords:** HIV, ubiquitin, proteasome, restriction factors, TRIM5α, March8, APOBEC, SAMHD1, BST2/Tetherin

## Abstract

The ubiquitin-proteasome system (UPS) ensures regulation of the protein pool in the cell by ubiquitination of proteins followed by their degradation by the proteasome. It plays a central role in the cell under normal physiological conditions as well as during viral infections. On the one hand, the UPS can be used by the cell to degrade viral proteins, thereby restricting the viral infection. On the other hand, it can also be subverted by the virus to its own advantage, notably to induce degradation of cellular restriction factors. This makes the UPS a central player in viral restriction and counter-restriction. In this respect, the human immunodeficiency viruses (HIV-1 and 2) represent excellent examples. Indeed, many steps of the HIV life cycle are restricted by cellular proteins, some of which are themselves components of the UPS. However, HIV itself hijacks the UPS to mediate defense against several cellular restriction factors. For example, the HIV auxiliary proteins Vif, Vpx and Vpu counteract specific restriction factors by the recruitment of cellular UPS components. In this review, we describe the interplay between HIV and the UPS to illustrate its role in the restriction of viral infections and its hijacking by viral proteins for counter-restriction.

## 1. Introduction

The human cell is in a continuous arms race with various viruses. This has led to the coevolution of cellular restriction factors on the one hand and viral proteins for counter-defense on the other hand. Restriction factors are generally induced as a result of an interferon response—they use unique mechanisms to impair specific steps of the replication cycle and they exhibit a dominant and autonomous effect. In this continuous fight, the ubiquitin-proteasome system (UPS) plays a central role on the cellular as well as on the viral side. The cell expresses restriction factors, some of which are themselves components of the UPS, targeting viral proteins for degradation and thereby inhibiting some crucial steps of the viral life cycle. However, viruses have evolved to use the UPS to their own benefits, subverting components of the UPS to degrade restriction factors, thereby protecting themselves from the cellular defense machinery to allow their dissemination. In this review, we will describe the mechanisms by which the human immunodeficiency viruses (HIV-1 and 2) use and subvert the UPS in the continuous battle between cellular defense and viral counter-defense.

## 2. The Ubiquitin-Proteasome System

**Figure 1:**
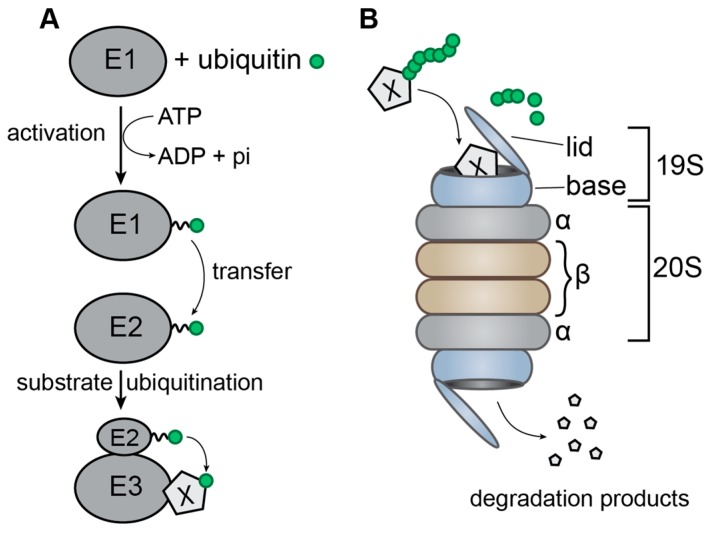
Schematic representation of the ubiquitin-proteasome system. (**A**) Transfer of ubiquitin from the ubiquitin-activating enzyme E1 to the ubiquitin-conjugating enzyme E2 followed by its transfer onto the target protein X by the ubiquitin ligase E3. The broken line symbolizes the thiol-ester bond; (**B**) the 26S proteasome, composed of the 20S barrel and two 19S lids. The ubiquitinated target protein X is recognized by one of the lids and translocated through the barrel where it is degraded by the proteases located on the inside of the β-rings.

The ubiquitin-proteasome system (UPS) is an important pathway in the cell, ensuring regulation of the protein pool in the cytoplasm as well as in the nucleus. The UPS is constituted by three main components: the proteasome holoenzymes, several ubiquitin ligases and a large variety of deubiquitinating enzymes (DUBs) [1]. Ubiquitin is a ubiquitously expressed and well-conserved eukaryotic peptide of 76 amino acids, which can be conjugated to proteins, mainly on their lysine residues. The addition of a single ubiquitin or small ubiquitin chains is involved in many regulatory functions, whereas poly-ubiquitination at lysine 48 (K48), corresponding to the addition of chains exceeding four ubiquitins, serves as a signal for degradation [2,3,4,5]. Ubiquitination is dependent on the ubiquitin machinery: the ubiquitin-activating enzyme E1 first forms a high-energy thiol-ester link with ubiquitin in an ATP dependent manner; ubiquitin is then transferred onto a thiol group of the ubiquitin-conjugating enzyme E2; finally, the ubiquitin ligase E3 transfers ubiquitin onto a lysine of its substrate (Figure 1A) [6,7]. In humans, there are two E1 enzymes, around 40 different E2 enzymes, which primarily determine the type of ubiquitin chain that is added, as well as over 700 different E3 ubiquitin ligases, which ensure targeting of various substrates and can be separated into two main families: HECT (Homologous to E6-AP Carboxyl Terminal) and RING (Really Interesting New Gene) ubiquitin ligases. DUBs are equally important for maturation, regulation and recycling of ubiquitin [2,3,4,5].

A protein can be conjugated to different types of polyubiquitin chains, depending on which of the seven lysine residues of ubiquitin is used to link ubiquitin moieties in the chain. Proteins ubiquitinated by K48-linked chains are mainly destined for proteasomal degradation [8,9]. The 20S proteasome is a barrel-shaped structure composed of four rings: two outer rings composed of seven α-subunits and two inner rings composed of seven β-subunits, which carry the protease activity on the inside of the ring. The 26S proteasome is formed by association of a 20S proteasome with two 19S lids, which ensure specific recognition of ubiquitinated substrates, recycling of ubiquitin through deubiquitination, unfolding of the target protein and translocation through the 20S barrel (Figure 1B) [2,10,11,12,13,14]. While the proteasome represents the main degradation mechanism used in cells, some membrane-associated proteins are degraded by the endo-lysosomal pathway, which can be induced by mono-ubiquitination or K63-linked polyubiquitination. In this pathway, ubiquitinated membrane proteins are endocytosed and are then recognized by the endosomal sorting complexes required for transport (ESCRT), which mediate invagination of the endosomal membrane surrounding the ubiquitinated protein. The core ESCRT machinery consists of the ESCRT-I, ESCRT-II and ESCRT-III complexes, ALIX (Apoptosis-Linked gene 2-Interactiong protein X) and VPS4 (Vacuolar Protein Sorting-associated 4). This results in the formation of small vesicles inside the endosome, thereby generating what is called a multivesicular body (MVB). This MVB can then fuse to the lysosome, where the internal vesicles and their associated proteins are degraded [3,15,16,17].

The UPS plays a central role in many viral infections (reviewed in [18,19,20]), with five main modes of action on the viral life cycle:

(1) Some cellular E3 ubiquitin ligases recognize viral proteins and induce their ubiquitination, which can have a positive effect on viral replication. For instance, ubiquitination of the p6 domain of the HIV-1 Gag polyprotein is important for the interaction of p6 with the ESCRT machinery. However, the mono-ubiquitination of lysine residues within the p6 domain (K27 and K33) does not seem to be sufficient to facilitate budding of new virions, the latter being also dependent on the cumulative ubiquitination of NC-p2 (NucleoCapsid-peptide 2) domain [21,22,23,24]. Ubiquitination of the HIV-1 accessory protein Tat by cellular E3 ligases stimulates transcription of viral RNA [25,26].
(2) Ubiquitination of viral proteins can also induce their degradation, thereby blocking the viral life cycle. This is a strategy used by certain restriction factors. The polymerase PB1 (Protein Binding 1) of the *Influenza A virus* (IAV) for example is ubiquitinated (K48-linked ubiquitin) by the cellular E3 ubiquitin ligase TRIM32 (TRIpartite Motif-containing protein 32), followed by its degradation by the proteasome [27]. This seems to be a general mechanism as PB1 proteins derived from various IAV serotypes (H1N1 (Hemagglutinin 1 Neuraminidase 1), H3N2, H5N1 or H7N9) associate with TRIM32 in multiple cell types and this suggests that PB1 has not yet adapted to avoid TRIM32 targeting [28]. The *Human herpesvirus type I* (HSV-1) capsid protein Vp5 has also been shown to be degraded by the ubiquitin proteasome system, leaving the viral genome exposed to innate immune sensors [29]. Interestingly, TRIM5α was reported to inhibit HSV-1 and -2 replication at an early stage of the infection cycle [30], suggesting a role for this or related protein in cytosolic sensing of herpesvirus capsids.
(3) Certain viruses have evolved to recruit the cellular E3 ligases to induce the degradation of cellular proteins that might have harmful effects on the viral life cycle. For instance, the protein E6 of *Human papillomavirus* (HPV) recruits the cellular E3 ubiquitin ligase E6-AP to induce ubiquitination and degradation of p53, thereby allowing viral replication [31,32]. The NSP1 (Non-Structural RNA binding protein 1) protein of *Rotaviruses* subverts the Skp1-Cul1-Fbox (SCF) E3 ligase to induce the ubiquitination and degradation of β-TrCP (β-Transducin repeat Containing Protein). β-TrCP is by itself a substrate adaptor of an E3 ligase and its degradation leads to accumulation of the NF-ϰB inhibitor IϰB, resulting in inhibition of the NF-ϰB induced antiviral responses [33,34]. These mechanisms are important for HIV replication and will be detailed in Section 5.
(4) Other viruses directly encode their own E3 ligases. *Kaposi sarcoma herpesvirus* (KSHV) protein K3 and K5 (RING-CH family of ligases) ubiquitinate MHC-I (Major Histocompatibility Complex I), resulting in its down-regulation from the cell surface through a clathrin-dependent sorting pathway to an endolysosomal compartment [35,36]. This endolysosomal sorting requires K63-linked instead of K48-linked polyubiquitin chains [19]. Another well-known example is the ICP0 protein (Infected Cell Protein 0) of HSV-1, an E3 ubiquitin ligase which induces the degradation of the ND10 (Nuclear Domain 10) nuclear body components PML (Promyelocytic Leukemia Protein) and Sp100 through the UPS, thereby avoiding antiviral sensing [37,38]. ICP0 has also been shown to have a RING-independent E3 ligase activity that polyubiquitinates the E2 enzyme cdc34. ICP0 influences many cellular pathways and is required for the activation of most viral and many cellular genes, for reactivation from latency and suppression of innate immunity [19].
(5) Finally, ubiquitin modifications can be reversed by the isopeptide-bond specific proteolytic activity of DUBs. In addition to cellular DUBs, it has been reported that various virus families code their own DUBs (Coronavirus, Herpesvirus etc.) to evade host antiviral immune response and promote virus replication (for a recent review see [1]). For instance, in the herpesviridae family, a variety of DUBs play an important role in the virus life cycle (e.g., UL36USP (Ubiquitin Ligase 36 Ubiquitin Specific Protease) of HSV-1, tegument protein pUL48 of human cytomegalovirus (HCMV)). Regarding HIV-1, a recent study reported that several cellular DUBs (USP7 and USP47, Ubiquitin Specific Protease family) play an important role in its replication by regulating Gag processing and thus the infectivity of released virions and simultaneously the entry of Gag into the UPS and MHC-I pathway [39]. Moreover, this study showed that treatment with DUB inhibitors targeting USP47 causes a general Gag processing defect, indicating that USP47 interacts with Gag and prevents its entry into the UPS. Similarly, proteasome inhibitors have been shown to impact HIV-1 replication by reducing the release and maturation of infectious particles [40,41] or by suppressing its transcription [42]. Taken together, these studies suggest a potential antiretroviral activity of DUB and proteasome inhibitors.

The importance of the UPS in antiviral restriction will be discussed here using HIV as an example.

## 3. The HIV Life Cycle

**Figure 2:**
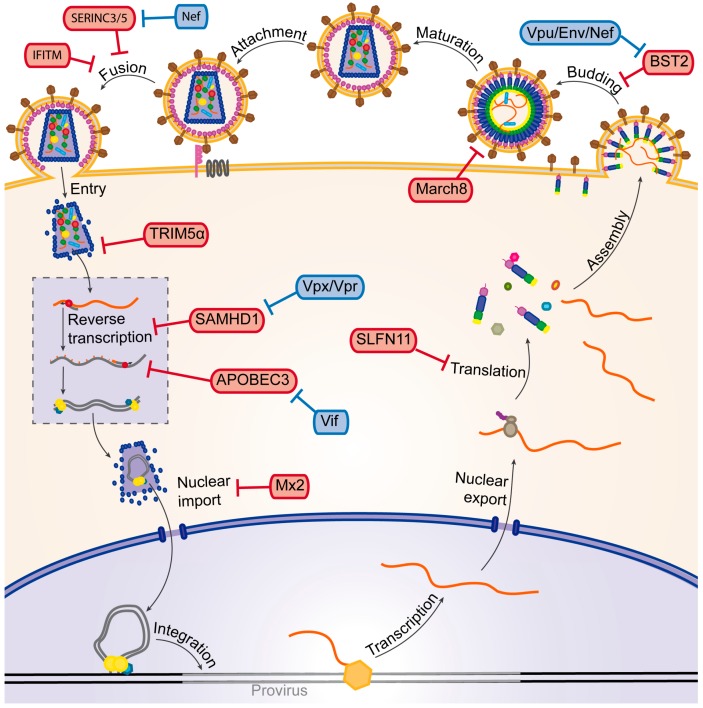
Schematic representation of the HIV-1 life cycle. The main HIV-1 restriction factors and the viral auxiliary proteins that counteract these factors (represented by T bars) are highlighted in red and blue boxes, respectively. See text for a description of the different steps of the life cycle.

HIV-1 and 2 are retroviruses of the genus *Lentivirus*. Their genome is composed of two (+) single stranded RNAs encoding the Gag, Pol and Env polyproteins, which correspond to the structural (matrix, capsid, nucleocapsid and p6), enzymatic (protease, reverse-transcriptase and integrase) and envelope (transmembrane and surface) viral proteins. In addition, the genome of these two viruses express two regulatory (Tat and Rev) and four auxiliary (Nef, Vpu/Vpx, Vpr and Vif) proteins, which regulate several steps in the viral life cycle [43,44]. The main difference between HIV-2 and HIV-1 is the lack of the Vpu protein in the former, which is replaced by Vpx [45]. Following viral attachment and entry into the target cell, the dimeric viral genomic RNA is partially uncoated and transported to the cell nucleus. Concomitantly, reverse transcription of the viral genomic RNA takes place to form the pre-proviral DNA, which is then integrated into the cellular genome. The integrated provirus mediates the synthesis of new full-length viral RNA (or unspliced RNA), which will be used as genomic RNA encapsidated into viral particles and as mRNA for structural and enzymatic proteins and mono- and multi-spliced viral mRNAs, which encode the viral envelope and the regulatory and auxiliary proteins in the infected cell. Finally, the components of the viral particle assemble at the plasma membrane, where new viral particles bud, maturate and disseminate to other host cells in the infected organism (Figure 2) [43,44,46,47,48,49].

During its life cycle, HIV is subjected to different cellular restriction factors (Figure 2), the first line of defense of cellular immunity. The newly discovered SERINC3 (SERine INCorporator 3) and SERINC5 proteins target the very beginning of the viral life cycle by inhibiting correct fusion of the viral envelope with the plasma membrane, thereby preventing the virus from entering into a new host cell [50,51]. IFITM (InterFeron-Induced TransMembrane) proteins 1, 2 and 3 also target the viral entry into the cell by inhibiting viral fusion with target cells. The exact mechanism of restriction is yet a matter of debate, as well as whether IFITM incorporation in virions or its expression in target cells is responsible for the antiviral effect. IFITM proteins might act on Env to inhibit its functions in viral fusion and it has been shown that some mutations in the Env protein can indeed confer resistance to IFITM restriction [52,53,54,55,56,57]. Once the virus has entered the cell, TRIM5α (TRIpartite Motif-containing protein 5α) can inhibit the early steps of the viral life cycle in a species-specific manner by accelerating viral uncoating [58,59,60]. The viral capsid protein also seems to be the target of Myxovirus resistance 2 (Mx2/B), a restriction factor that inhibits nuclear import and subsequent integration of the provirus through an unknown mechanism.

Some mutations in the capsid protein have been shown to confer resistance to Mx2 and particularly some mutations located at the site of interaction with cyclophilin A, an important host factor for HIV-1 infectivity [61,62,63,64,65,66]. SAMHD1 (Sterile Alpha Motif and Histidine Aspartate domain-containing protein 1) also targets the early phase of viral infection: this deoxynucleotide-triphosphohydrolase inhibits reverse transcription by depleting the pool of cellular dNTPs (deoxy Nucleotide TriPhosphates) [67,68]. During reverse transcription of the viral RNA, the restriction factor APOBEC3G (APOlipoprotein B mRNA Editing enzyme, Catalytic polypeptide-like 3G, or A3G) and other factors from the APOBEC3 family, can induce G to A hypermutations, which prevent production of functional viral proteins [69,70,71]. The amount of viral proteins that are produced in an infected cell can be limited by Schlafen11 (SLFN11). Due to the bias of HIV-1 towards A/U rich codons, the virus stimulates production of corresponding tRNAs by the cell to increase viral translation, a mechanism that seems to be partly counteracted by SLFN11, which binds tRNAs in a codon-specific manner [72,73,74]. The final steps in the viral life cycle can be targeted by Tetherin/BST2 (Bone marrow STromal antigen 2), which inhibits release of new viral particles from the host cell [75,76,77] and March8 (Membrane-Associated RING-CH 8 protein), which decreases incorporation of envelope proteins into newly produced virions, thereby decreasing their infectivity [78]. Two of these restriction factors, TRIM5α and March8, use the UPS to exert their restricting activity.

HIV is able to counteract restriction factors using its accessory proteins (Figure 2): Nef prevents SERINC5 incorporation into virions by mediating its relocalization to late endosomes through interaction with the clathrin adaptor AP-2 [50,79]. Vif counteracts A3G by inducing its proteasomal degradation as well as by reducing its transcription and translation [69,80,81,82]. Vpx (and Vpr of certain Simian Immunodeficiency Virus (SIV) strains) counteracts SAMHD1 by inducing its proteasomal degradation [67,83,84]. Vpu (Env for HIV-2 and Nef or Vpu for SIV) counteracts BST2/Tetherin by sequestering it away from sites of viral budding [76,77,85]. Amongst these accessory proteins, Vif, Vpx and Vpu hijack the UPS to exert their counter-defense. In the following section, we will discuss in detail the restriction factors as well as the viral proteins which use the UPS for their respective mechanisms.

## 4. Cellular Factors Mediating Viral Restriction Using the UPS

### 4.1. TRIM5α

**Figure 3:**
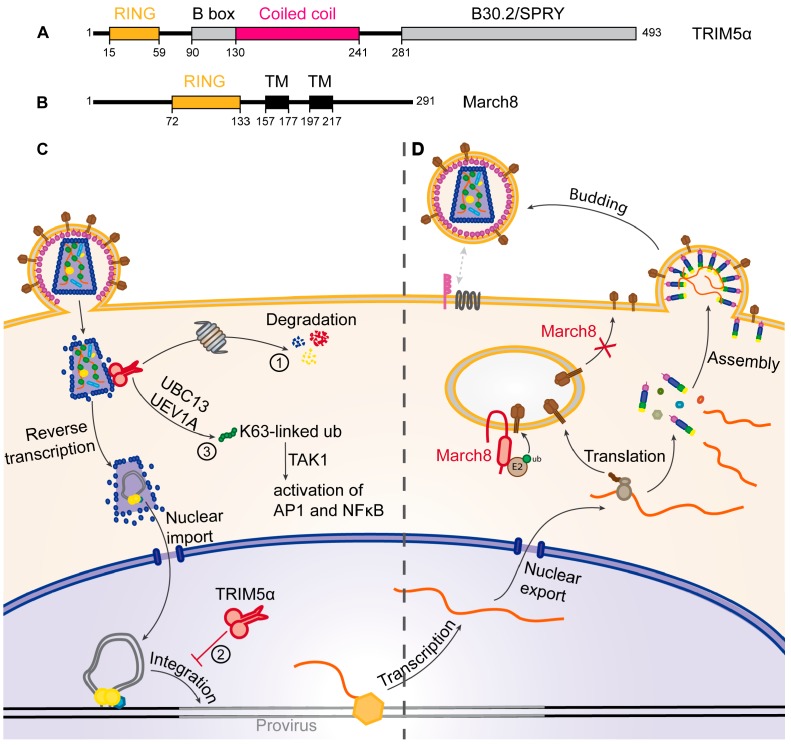
Restriction of HIV by TRIM5α and March8. (**A**,**B**) Schematic representation of the main domains of (**A**) the TRIM5α and (**B**) March8 proteins. Black boxes correspond to transmembrane domains (TM). Amino acid positions of the beginning and end of the domains as well as the total length of the proteins are indicated; (**C**) mechanism of TRIM5α restriction. The dimeric TRIM5α (red) recognizes the viral capsid and ① induces the proteasomal degradation of the capsid (blue), the integrase (yellow) and itself, leading to premature decapsidation of viral RNA. ② TRIM5α also blocks integration of the provirus (red T bar) and ③ induces activation of AP1 and NFκB pathways; (**D**) March8 (red) mediates intracellular retention of envelope proteins (Env, brown), leading to reduced Env incorporation into virions, thereby decreasing infectivity.

One example of the cell using the UPS to restrict HIV is TRIM5α, an E3-ubiquitin ligase that interacts with the viral capsid after its entry into the cell. TRIM5α mediates a species-specific block: HIV-1 is restricted by the TRIM5α proteins of old world monkeys like rhesus or cynomolgous monkeys, while the TRIM5α of human or new world monkeys have no or only a very weak effect on HIV-1 [59,60,86,87]. TRIM5α thereby constitutes one of the factors responsible for the interspecies barrier. The restriction of HIV-1 by TRIM5α is mediated by the interaction of the TRIM5α SPRY (SPIa and RYanodine Receptor) domain (Figure 3A) with the viral capsid in the cytoplasm of newly infected cells [59]. This interaction leads to premature decapsidation of the viral core. Moreover, viral capsid and integrase proteins are degraded (Figure 3C①) and the reverse transcription of the viral genome is inhibited in the presence of a restricting TRIM5α. These effects seem to be mediated by the UPS, since treatment with proteasome inhibitors restores a normal decapsidation rate and reverse transcription. It has also been shown that the proteasome co-localizes with TRIM5α and viral cores in the cytoplasm [88,89]. TRIM5α is also degraded by the proteasome but only in the presence of susceptible viral cores [90], suggesting that TRIM5α recruits the proteasome to the viral cores and induces their degradation. This mechanism seems to be mediated by the E3-ubiquitin ligase activity of TRIM5α, through its RING domain (Figure 3A) [58,91]. Nevertheless, TRIM5α inhibits integration of the proviral DNA independently of the proteasome, suggesting that TRIM5α uses an additional, yet uncharacterized, strategy to block viral infection (Figure 3C②) [92,93]. Finally, the association of TRIM5α with the viral capsid enhances its E3-ubiquitin ligase activity, which, in conjunction with the E2 enzyme UBC13/UEV1A (UBiquitin-Conjugating enzyme 13/Ubiquitin-conjugating Enzyme Variant 1A), leads to the synthesis of free K63-linked ubiquitin chains, thus stimulating TAK1 (Transforming growth factor β-Activated Kinase 1) and finally activating AP1 and NF-ϰB signaling (Figure 3C③) [94,95]. This indicates that TRIM5α, in addition to its direct antiviral activity, also functions as a sensor that induces a general antiviral state of the cell.

### 4.2. March8

March8 has recently been identified as a restriction factor of HIV-1, expressed by differentiated myeloid cells like monocyte derived macrophages and dendritic cells [78]. March8 significantly reduces infectivity of virions produced from March8-expressing cells by decreasing the number of Env-proteins incorporated into budding virions. March8 is a transmembrane E3-ubiquitin ligase, possessing an N-terminal, cytoplasmic RING domain (Figure 3B), known to downregulate multiple cellular proteins from the plasma membrane by ubiquitination followed by degradation in the endo-lysosomal pathway [96,97,98,99]. In the case of HIV-1 restriction, it has been shown that March8 interacts with Env and causes its downregulation from the cell surface. The RING-domain of March8 is necessary for this mechanism, suggesting that ubiquitination plays a role. However, Env does not seem to be degraded in the endo-lysosomal pathway like cellular proteins targeted by March8 but seems rather to be retained in intracellular compartments. March8 thus sequesters Env away from HIV-1 budding sites, thereby reducing Env incorporation into newly formed virions, making them less competent for infection of new target cells (Figure 3D) [78].

## 5. Counteraction of Restriction Factors by Viral Auxiliary Proteins Using the UPS

### 5.1. Vif

**Figure 4:**
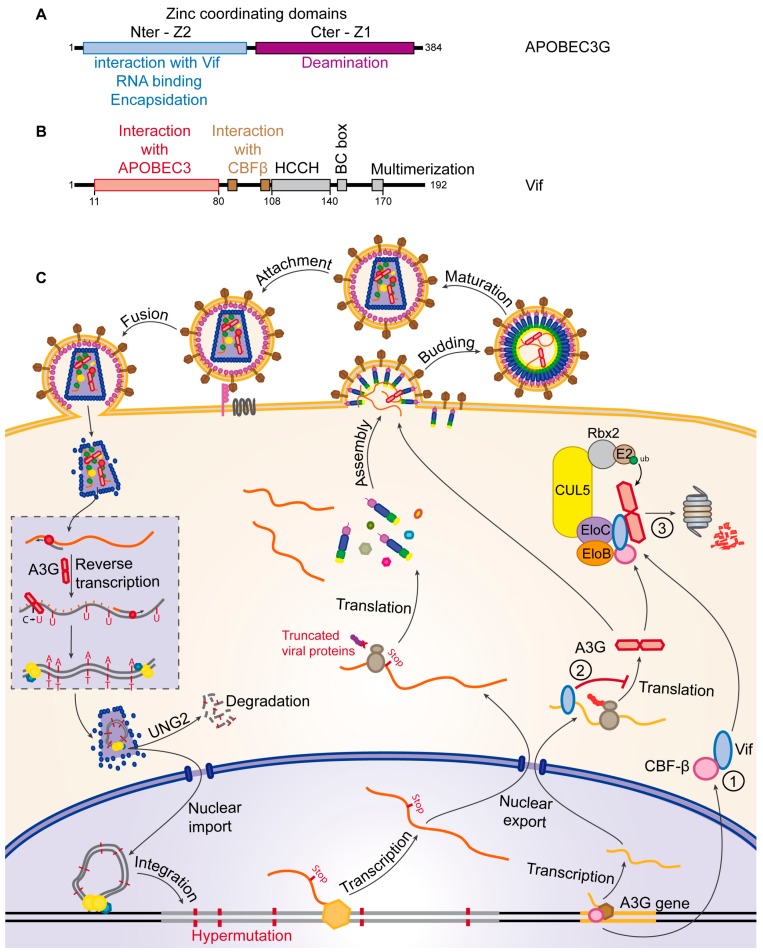
Restriction of HIV by APOBEC3G and counteraction by Vif. (**A**,**B**) Schematic representation of the main domains of (**A**) the APOBEC3G and (**B**) Vif proteins. Amino acid positions of the beginning and end of the domains as well as the total length of the proteins are indicated; (**C**) the mechanism of APOBEC3G restriction and Vif counteraction. APOBEC3G (red) is incorporated into virions and induces hypermutations of the provirus leading either to its degradation or production of truncated viral proteins. Vif (blue) decreases A3G transcription ①, inhibits its translation ② (Red T bar) and induces its degradation by the proteasome ③.

The family of Apolipoprotein B mRNA-editing enzyme, catalytic polypeptide-like 3 (APOBEC3/A3) proteins, is a family of 7 cytosine deaminases (A3A to A3H) which induce transition of cytosine to uracil on single-stranded DNA, with a preferential recognition of CC sequence motifs by A3G and TC motifs by the others [100,101,102]. A3G (Figure 4A) has been the first member of this family to be identified as a potent antiviral factor. It is incorporated into budding HIV virions and is thereby carried over into the next infected cell [69]. During reverse transcription of the viral genomic RNA, the single stranded negative sense DNA is sensitive to the cytosine-deaminase activity of A3G, leading to C to U transitions [70,71]. These mutations can either be recognized by uracil DNA glycosylases, like the virion-associated UNG2 (Uracyl N-Glycosylase 2), leading to the degradation of the provirus by abasic site endonucleases [103], or they can be conserved in the provirus. Due to the sequence preference of A3G, these mutations very frequently introduce new stop codons in the viral genome, thus leading to the expression of non-functional mutated or/and truncated viral proteins (Figure 4C). HIV-1 counteracts A3G with its Vif protein, which prevents A3G incorporation into virions by inducing its degradation through the proteasome [80]. To do so, Vif recruits an SCF-like E3-ubiquitin ligase, composed of Cullin5, Rbx2, Elongin B and C. In this complex, Vif possesses the role of a substrate adaptor, directly interacting with A3G through its N-terminal domain (Figure 4B), thereby recruiting it for ubiquitination (Figure 4C③) [104].

The recruitment of Cullin5 is mediated by the zinc-binding domain of Vif [105] and Cullin5 in turn recruits the E2-ubiquitin-conjugating enzyme Rbx2. The recruitment of Elongin B and C is mediated by the BC-box domain of Vif (Figure 4B), which can be negatively regulated by phosphorylation. In this complex, not only A3G but also Vif is ubiquitinated, which might contribute to the transport of A3G to the proteasome [106]. The cellular protein HDAC6 (Histone Deacetylase 6) has been shown to play a role in this process, by inducing Vif degradation through autophagosomes as well as by protecting A3G from ubiquitination and degradation [107]. The expression level of Vif is also regulated by Mdm2 (Mouse double minute 2 homolog), an E3-ubiquitin ligase that can induce the ubiquitination of Vif and its proteasomal degradation [108]. CBF-β (Core Binding Factor β), a co-factor of the RUNX transcription factor family, is recruited by Vif and ensures its stability by inhibition of Mdm2 binding [109]. CBF-β is also necessary to allow assembly of the SCF-like E3-ubiquitin ligase mediated by Vif, resulting in the inability of Vif to induce ubiquitination and degradation of A3G in the absence of CBF-β [110,111]. Moreover, by sequestering CBF-β in the E3-ubiquitin ligase complex, Vif indirectly causes a decrease in A3G transcription as the *A3G gene* is regulated by the RUNX transcription factor family, which requires CBF-β as cofactor (Figure 4C①) [81]. Degradation of A3G through the UPS has been known for a long time as the main mechanism for HIV-1 to counteract cellular restriction; however it has been shown that Vif can also inhibit A3G translation [82,112,113] and this inhibition significantly contributes to the counteraction mechanism (Figure 4C②) [82,112,113]. While A3G is the main member of the A3-family that efficiently restricts HIV, A3D, F and H also showed a restricting activity towards HIV-1 in the absence of Vif, even though to a lesser extent than A3G [114]. Vif is also able to recruit these A3 proteins by different motifs of its N-terminal domain (Figure 4B), thus inducing their degradation by the proteasome similarly to A3G [115,116,117].

### 5.2. Vpx

**Figure 5:**
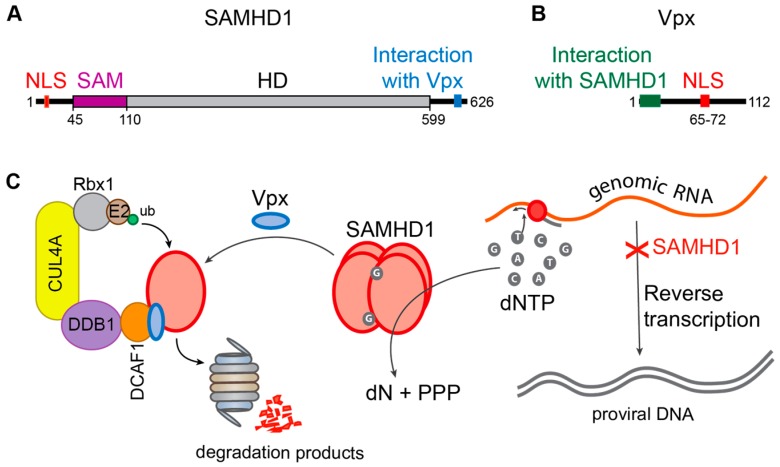
Restriction of HIV by SAMHD1 and counteraction by Vpx. (**A**,**B**) Schematic representation of the main domains of (**A**) SAMHD1 and (**B**) Vpx. The nuclear localization signal (NLS) is indicated in red. Amino acid positions of the beginning and end of the domains as well as the total length of the proteins are indicated; (**C**) the mechanism of SAMHD1 restriction and Vpx counteraction. Tetrameric SAMHD1 (red) hydrolyzes dNTPs, leading to a block of reverse transcription of the viral genome. Vpx (blue) induces SAMHD1 ubiquitination followed by its degradation by the proteasome.

Sterile alpha motif and histidine-aspartate domain-containing protein 1 (SAMHD1, Figure 5A) is a dGTP-regulated deoxynucleoside-triphosphohydrolase that catalyzes the hydrolysis of dNTPs to deoxynucleosides and inorganic triphosphate [118,119]. In non-cycling myeloid cells as well as in resting CD4^+^ T cells, this restriction factor causes a block in the early steps of the HIV-1 life cycle [67] by depleting the intracellular pool of dNTPs [68], which leads to abortion of the viral genomic RNA reverse transcription and accumulation of defective viral cDNA (Figure 5C) [120]. This block strongly affects infectivity of HIV-1 in these cell types but has no effect on HIV-2 infectivity [121]. Indeed, HIV-2 possesses the viral protein X (Vpx, Figure 5B) which alleviates the post-entry block mediated by SAMHD1 by inducing its degradation by the proteasome. Vpx has been found to recruit the CUL4A-DDB1-DCAF1(DDB1 and CUL4 Associated Factor 1) E3 ubiquitin ligase through a direct interaction with its substrate recognition protein DCAF1 (DDB1 and CUL4 Associated Factor 1) [122] while also interacting with the C-terminal domain of SAMHD1, thereby loading SAMHD1 onto the E3 complex and inducing its ubiquitination followed by its proteasomal degradation (Figure 5C). The nuclear localization of SAMHD1 is required for its Vpx-induced proteasomal degradation, suggesting the nuclear UPS is important in this mechanism [123,124]. Degradation of SAMHD1 leads to an increase in cellular dNTP levels and the efficiency of proviral DNA synthesis [120]. In this manner, the Vpx protein allows HIV-2 to efficiently infect human dendritic and myeloid cells and it significantly increases the infection by HIV-1 [83]. Vpx therefore seems to be an important protein for viral replication, however it is present exclusively in HIV-2 and some SIV strains.

In these lineages, the *Vpx gene* has evolved from Vpr which is present in all HIV and SIV strains and whose main function is the induction of cell cycle arrest [125,126,127,128,129]. Vpx and Vpr share many similarities, like for example their interaction with the same CUL4A E3 ubiquitin ligase [122,125]. Interestingly, the Vpr protein of some SIV strains has been shown to induce proteasomal degradation of SAMHD1, thereby compensating for the lack of Vpx. Indeed it seems that the ability to degrade SAMHD1 has first been acquired by the Vpr protein in certain lentiviral strains before the evolution of a separate *Vpx gene* which has subsequently conserved the function of SAMHD1 antagonism [84,130]. Nevertheless, many lineages, like HIV-1 for example, lack an anti-SAMHD1 activity. HIV Interestingly, SAMHD1 seems to be regulated in cells by phosphorylation mediated by CDK6-(Cyclin-Dependent Kinase 6) dependent CDK2, which links its activity to cell cycle control. Indeed, SAMHD1 is phosphorylated in cycling cells which blocks its activity as a dNTP hydrolase [131]. This correlates with the permissiveness of cycling cells for HIV-1 infection as opposed to non-cycling cells. Moreover, HIV infection is made possible despite the lack of a viral factor counteracting SAMHD1 by different cellular proteins: CD81 for example has recently been shown to favor HIV-1 infection by interacting with SAMHD1 and stimulation of its proteasome-dependent degradation [132]. Cyclin L2 also induces SAMHD1 proteasomal degradation through interaction with SAMHD1 and DCAF1, a mechanism interestingly similar to the one used by Vpx [133].

### 5.3. Vpu

**Figure 6:**
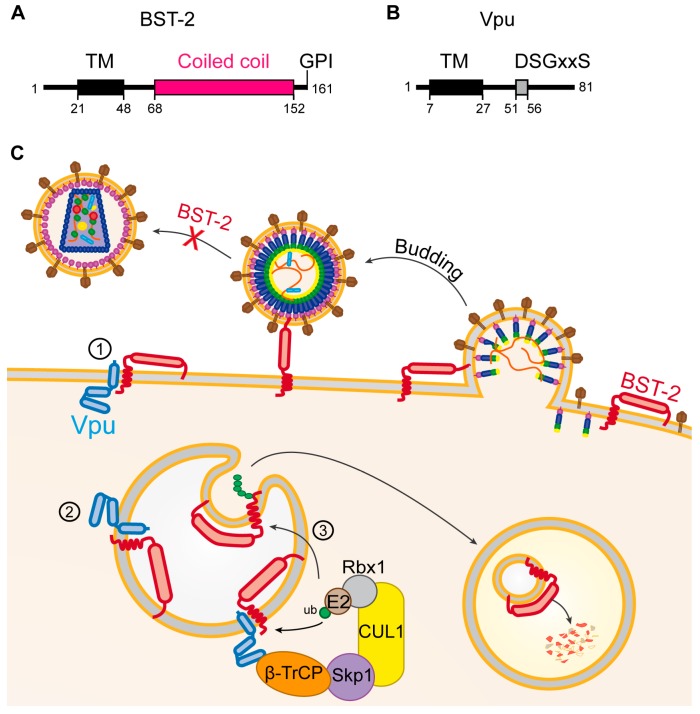
Restriction of HIV by BST-2 and counteraction by Vpu. (**A**,**B**) Schematic representation of the main domains of (**A**) the BST-2 and (**B**) Vpu proteins. Black boxes indicate transmembrane domains (TM). The glycosyl-phosphatidylinositol (GPI) modification at the C-terminal end of BST-2 is indicated. Amino acid positions of the beginning and end of the domains as well as the total length of the proteins are indicated; (**C**) mechanism of BST-2 restriction and Vpu counteraction. BST-2 tethers virions to the plasma membrane, thereby hindering their dissemination. Vpu sequesters BST-2 away from virion budding sites either at the plasma membrane ① or in intracellular compartments ②. Vpu can also induce BST-2 degradation in the endo-lysosomal pathway ③.

In the absence of Vpu, newly formed virions remain tethered to the plasma membrane of their host cell after budding and are eventually endocytosed and degraded [75]. The cellular restriction factor responsible for the block of virion release is Tetherin/BST-2. BST-2 is found as a disulfide-bond-linked dimer which is anchored into the plasma membrane by two domains: a transmembrane domain close to its N-terminus and an extracellular C-terminal glycosyl-phosphatidylinositol (GPI)-anchor (Figure 6A) [134]. These two domains mediate virion-tethering to the host cell, one remaining in the plasma membrane and the other one being inserted into the viral envelope (Figure 6C). It has been shown that this tethering involves approximately a dozen of BST-2 dimers and that among the two membrane-associated domains, the GPI-anchor is preferentially incorporated into budding virions [135]. The extracellular domain of BST-2 thereby acts like a molecular ruler, maintaining the virus at a constant distance of the plasma membrane, preventing it from disseminating to other target cells [134].

The viral protein Vpu counteracts BST-2 by direct interaction of their transmembrane domains embedded in the plasma membrane [136]. The exact mode of action of Vpu is still a matter of debate, but it seems clear now that Vpu sequesters BST-2 away from virion budding sites, thereby preventing it from incorporation into the viral envelope (Figure 6C①) [77,85,137,138]. Several studies have shown that in the presence of Vpu, newly synthesized BST-2 is sequestered in intracellular compartments, particularly the trans-golgi-network (Figure 6C②). This finally results in the downregulation of surface levels of BST-2, thereby allowing normal rates of virion release in the presence of Vpu [77,85,137,138]. BST-2 is constitutively regulated by ubiquitination and lysosomal degradation mediated by the cellular E3 ubiquitin ligases March8 and NEDD4 (Neural precursor cell Expressed Developmentally Down-regulated protein 4) [139]. It is still a matter of debate however, whether Vpu also uses the endo-lysosomal system for BST-2 counteraction. The E3-ubiquitin ligase adaptor β-TrCP is known to be recruited by the cytoplasmic DSGxxS motif of Vpu (Figure 6B) [140], which might lead to ubiquitination of BST-2 followed by its degradation in the endo-lysosomal system (Figure 6C③) [137,141].

Even though the interaction of Vpu with β-TrCP, as well as the capacity of β-TrCP to recruit an E3-ubiquitin ligase seem to be required for BST-2 counteraction by Vpu [137,142,143], conflicting data have also been reported [144,145,146]. Certain components of the autophagy pathway, as well as clathrin adaptors AP-1 and 2 and components of the ESCRT system might also be involved in the downregulation of BST-2 by Vpu, which would corroborate transport of BST-2 in the endosomal system [137,147,148,149]. However, degradation of BST-2 might not be absolutely required for viral counteraction of BST-2, since Vpu is capable of intracellular sequestration of BST-2 independently of its degradation [85,138]. The guanylate binding protein 5 (GBP5) has very recently been discovered as a new restriction factor of HIV-1 infection, that interferes with viral Env proteins, thereby decreasing infectivity of produced virions [150,151]. As Vpu and Env are expressed from the same transcript by leaky scanning, the loss of Vpu expression can in this case lead to an increase of Env expression, as observed in the macrophage tropic AD8 isolate [152], allowing the virus to partly overcome GBP5 restriction. Surprisingly, such Vpu mutants seem to occur frequently despite the presence of BST-2. HIV-2 and SIV are also counteracted by BST-2 proteins expressed by their respective host species in a species-dependent manner, but some of them lack Vpu to counteract this mechanism. It has been shown that the HIV-2 Env protein can enhance virion release in the presence of BST-2 thereby substituting for Vpu [153,154]. Certain SIV strains, like SIVagm, SIVblu and SIVmac also lack the *Vpu gene* and rely on the accessory protein Nef to counteract BST-2. Other SIV strains like SIVmon, SIVmus, SIVgsn and SIVden express Vpu and use it to counteract BST-2. Even though SIVgor and SIVcpz express Vpu, Nef seems to take over the role of BST-2 counteraction. This gives interesting clues about the evolution of HIV and SIV strains [155,156,157].

## 6. Other Cellular Proteins Targeted by the Hijacked UPS

The UPS is hijacked by HIV and plays an important role for the viral defense against multiple cellular restriction mechanisms. Apart from restriction factors, several other cellular proteins can also be targeted by HIV through the UPS. The viral auxiliary protein Vpu for example possesses the ability to associate with the CUL1-Skp1 E3 ubiquitin ligase through interaction with its substrate receptor β-TrCP. This association not only seems to play a role in the counteraction of BST-2 but has also been shown to induce degradation of the HIV receptor CD4. Indeed, Vpu induces CD4 ubiquitination followed by its extraction from the Endoplasmic Reticulum (ER) [140,158,159,160,161,162]. The mechanism used by Vpu to induce CD4 depletion involves the cellular ER-associated degradation (ERAD) pathway, which operates as a quality control mechanism to dispose of unwanted ER membrane proteins into the cytosol for subsequent proteasomal degradation. The dislocation of protein from the membrane is achieved by the recruitment of the VCP-UFD1L-NPL4 (Valosin-containing protein-Ubiquitin fusion degradation protein 1-Nuclear protein localization protein 4) complex through recognition by UFD1L of K48-linked poly-ubiquitin chains on the CD4 cytosolic tail. Interestingly, the degradation of CD4 depends also on ubiquitination of serine/threonine residues [140,158,159,160,161]. The ATPase activity of VCP then drives dislocation of CD4 from the ER membrane into the cytosol and eventually its degradation in proteasomes. The multiple levels at which Vpu acts to prevent export of CD4 from the ER underscore the importance of ensuring complete depletion of CD4 from the plasma membrane for progression of the infection [143,160,161,163]. Other targets of Vpu-induced ubiquitination and degradation include the cell surface glycoprotein ICAM-1 and the amino acid transporter SNAT-1, both involved in immune signaling [164,165].

It is well established that the viral auxiliary protein Vpr associates with the CUL4A-RING E3 ligase through interaction with its substrate recognition subunit DCAF1. This complex has been shown to induce ubiquitination followed by proteasomal degradation of the DNA glycosylase UNG2. Thereby Vpr reduces encapsidation of UNG2, ultimately contributing to the protection against the restriction factor A3G. UNG2 recognizes C to U mutations induced by A3G and generates abasic sites, leading to degradation of viral DNA. Indeed a virus lacking Vif can be partially rescued by Vpr-mediated reduction of UNG2 compared to viruses lacking both Vif and Vpr [166,167,168]. Moreover, it has recently been shown that Vpr can also induce the degradation of A3G itself through the UPS [169]. Vpr seems to also enhance HIV-1 production in macrophages by UPS-mediated degradation of the cellular protein Dicer, which is involved in RNA silencing [170]. The main function of Vpr known to date is the induction of a cell cycle arrest at the G2 phase. The association of Vpr with the CUL4A E3 ubiquitin ligase has been shown to be important for this process, although the exact mechanism is still unknown [125,126,127,128]. Cell cycle arrest seems to involve Vpr association with the SLX4-SLX1-MUS81-EME1 complex, leading to SLX4 (Structure-specific endonuclease subunit) activation and ultimately proteasomal degradation of MUS81 (Crossover junction endonuclease) and EME1 (Essential Meiotic Structure-Specific Endonuclease 1) [127,128]. Vpr also induces the degradation of multiple other cellular proteins such as the DNA translocase HLTF (Helicase-Like Transcription Factor) [171], the DNA replication factor MCM10 (Mini Chromosome Maintenance 10) [172], as well as the chromatin associated proteins ZIP (leucine Zipper), sZIP and class I HDACs (Histone Deacetylase 6) [173,174].

## 7. Conclusions

The UPS plays an important role in viral infections in general and especially in the process of viral restriction and counter-restriction. In this continuous battle between the virus and the cell, the UPS constitutes an efficient tool for both sides. Several HIV auxiliary proteins have evolved the ability to interact with components of the UPS, subverting it for its own means. This allows the targeting of a multitude of different cellular proteins through a single platform. This strategy is not limited to HIV, but is used by a plethora of different viruses to ensure various aspects of their life cycles. Overall, the specific degradation of certain cellular proteins in the UPS allows viruses to generate a favorable environment for their own replication. The almost universal role of the UPS in counteraction of cellular restriction factors by HIV makes the UPS an interesting target for antiviral therapy. One of the main difficulties in therapy-design against HIV is the rapid evolution of the virus, which easily escapes therapeutic molecules by mutation of the targeted viral proteins. Targeting the human UPS represents a promising antiviral strategy because it would allow to avoid the escape through mutations [175,176]. A better knowledge on how the virus hijacks the UPS and which components are involved in viral replication is crucial in this attempt.
